# 关于我国血液系统疾病病理诊断的几点思考

**DOI:** 10.3760/cma.j.issn.0253-2727.2023.02.001

**Published:** 2023-02

**Authors:** 志坚 肖

**Affiliations:** 中国医学科学院血液病医院（中国医学科学院血液学研究所），国家血液系统疾病临床医学研究中心，实验血液学国家重点实验室，细胞生态海河实验室，天津 300020 National Clinical Research Center for Blood Diseases, State Key Laboratory of Experimental Hematology, Haihe Laboratory of Cell Ecosystem, Institute of Hematology & Blood Diseases Hospital, Chinese Academy of Medical Sciences & Peking Union Medical College, Tianjin 300020, China

血液系统疾病是一门高度依赖实验诊断的学科，血液系统疾病诊断是一个以传统光镜下血细胞形态学分析为基础，结合流式细胞术免疫表型分析、细胞遗传学和靶向测序基因突变检测的整合诊断过程。诊断分型标准从纯粹以细胞形态学为基础的FAB标准，到现今的细胞遗传学和分子学优先于细胞形态学的第五版造血淋巴组织肿瘤WHO分型和髓系肿瘤和急性白血病国际共识分型（ICC）[1]–[2]，随着新的技术，如全基因组测序（WGS）、单细胞空间组学等从实验室向临床转化，组学分型（genomic classification）将是未来的发展趋势[3]，因此，对血液系统疾病病理诊断提出了新的要求，也带来了新的挑战。笔者联系我国血液系统疾病病理学科现况，以及面临的问题和挑战，做了几点思考。

一、实验室自建检测（LDT）将成为血液病理诊断试验的主流

血液系统疾病的诊断分型和预后分层体系不断更新，而且，随着分子靶向药物、单（双）克隆抗体药物和CAR-T为代表的活细胞免疫治疗药物的开发和上市，在靶点（合适患者）筛选、疗效评估等伴随诊断（companion diagnostic, CD）需求日趋增多，现有的体外诊断（in vitro diagnostic, IVD）产品愈且不能满足临床需求。LDT是由实验室自行研发、验证且只在实验室内部使用诊断产品[4]，由于其具有开发周期短、从实验室向临床转化快等特点，因而，在精准诊治新时代愈显重要。血液系统疾病诊断试验中，流式细胞术免疫表型分析、荧光原位杂交（FISH）、RT（real-time）-PCR、第二代测序技术（NGS）等众多检测项目都以LDT模式为主，中国医学科学院血液病医院病理中心已开展的血液系统疾病诊断检测项目共361项，其中IVD产品96项，其他属LDT。

LDT是创新性体外诊断产品，要求在有条件的检验部门开展。近年，我国尽管出台了一些LDT相关政策，但现今在诸如LTD方法学性能和临床性能确认标准、开展LTD实验室的质量管理体系标准、LDT使用试剂和检测系统标准等尚待制定和（或）细化明确。笔者认为现阶段“有条件的检验部门”至少符合以下几点：①LDT正式应用于临床前，必须完成检测性能与临床性能评估，因此，开展LDT的检验部门应符合国家药监局发布的“体外诊断试剂临床试验指导原则”中对“临床试验机构和人员”的要求；②建立了完善的室内质控体系［如通过了ISO15189和（或）CAP认证的实验室］；③有一个富有LDT经验的团队，如开展NGS检测须有涉及生物信息分析、报告解读等多方面人才，此外，由于涉及流式细胞技术的检测项目缺乏严格意义的质控物和标准品，结果分析更多地依赖操作者的经验，需要有较高的独立分析、结果解释的专业能力。

二、血液病理专科医师培训是提高诊断试验质量的重要举措

1993年，卫生部印发“关于实施临床住院医师规范化培训试行办法的通知”，此后在全国各地逐步开展不同程度、不同水平的住院医师规范化培训的前期探索。2009年卫生部又颁布了“临床住院医师规范化培训试行办法”，至2020年，已经建立了住院医师规范化培训制度，要求所有新进医疗岗位的本科及以上学历临床医师，包括病理科医师，全部接受住院医师规范化培训。在我国，尚未启动和开展专科医师（包括血液病理专科医师）培训。

如何进行血液病理专科医师培训，起始阶段我们可以借鉴国外经验。培训内容应包括：①外周血细胞分析：了解自动血液分析仪的操作原理并能解释外周血细胞计数各项参数结果的临床意义。②骨髓/淋巴结形态学诊断：骨髓和外周血分类计数，并结合临床资料和其他实验室数据,根据细胞形态学发现做出诊断和鉴别诊断；了解各种组织抗原在鉴别血液淋巴系统疾病中的目的、效用和局限性。③流式细胞术免疫表型分析：了解流式细胞仪的操作原理，可能的错误来源和相应的纠正方法，流式细胞术实验室的质量控制和质量保证问题；能够在流式分析中识别正常和异常的细胞群，能够有效地将细胞形态学和表型发现整合到连贯且有用的报告中，并能对报告做出解读。④细胞遗传学/分子学：基本熟悉细胞遗传学和分子学在血液系统疾病诊断的作用，各种检测试验的优、缺点，可能的错误来源，能绘制分子病理学实验室的QC和QA流程图，为下一步分子病理学深入学习奠定一定的基础。⑤出凝血试验：熟悉每一项检验的标准操作程序（SOP）；熟悉血小板聚集检测项目的结果解释及其在获得性和遗传性血小板功能障碍疾病诊断中的作用；熟悉解释肝病、维生素K缺乏肝素效应、DIC、狼疮抗凝物、血管性血友病、血友病、阻断抑制剂和高凝状态的凝血曲线；熟悉血栓弹力图原理；了解因子V Leiden、凝血酶原变异突变和其他血栓形成的遗传或获得性遗传风险的诊断和意义。

通过血液专科病理医师培训后，首先，构建起了一个常见血液系统疾病的知识框架，特别是要熟悉各类疾病的诊断分型系统和诊断分型标准。其次，熟练掌握病理与临床的沟通，接收检验标本后在具体做分析前，主动去了解该患者涉及诊断的关键临床病史（如简要病史、家族史、治疗史等）、主要临床体征（包括有助诊断和鉴别诊断的阳性与阴性体征）、已知的辅助检查结果（血常规、影像检查等）和临床医师的拟诊诊断，分析起来就有了方向性。遇到疑难病例，应主动组织和参加临床-病理多学科讨论。再次，能独立出具结合包括细胞形态学、免疫表型分析、细胞遗传学和分子学的整合病理报告。最后，熟悉血液病理实验室的管理，特别是质量管理体系。

三、整合病理报告将是一种必然趋势

血液系统疾病病理分析方法常用的包括细胞形态学、免疫表型分析、细胞遗传学和分子学。细胞形态学除常规光镜下细胞形态学分析外，往往需结合细胞（组织）化学染色（如髓过氧化物酶染色、过碘酸希夫染色、氯乙酸AS-D萘酚酯酶染色、甲苯胺蓝染色等）、特殊染色（如铁染色、网状纤维染色等）、免疫细胞（组织）化学染色（如CD34、CD41a、CD61、CD117）和荧光原位杂交来加以综合判断，有时还需电子显微镜超微结构分析和免疫电镜检测。

由于不同的血液病在细胞形态学、免疫表型、遗传学及分子学等方面具有各自独特的特征，而不同的方法学在诊断中又具有各自的优、缺点，如血细胞形态学包括细胞形态学（外周血和骨髓涂片）和组织形态学（骨髓活检、淋巴结活检等），骨髓涂片主要用于评估各系别、每个系别各阶段细胞的比例和单个细胞形态细节，尤其是粒系、红系和单核系细胞的识别、区分及观察有无形态发育异常等，而骨髓活检则更侧重于骨髓增生程度，造血细胞的组织形态（分布及形态等）尤其是巨核细胞的分布和形态、非造血细胞、间质成分、骨小梁变化等内容的观察。两者互相补充、相辅相成，缺一不可，联合使用可提高疾病诊断率，减少误诊和漏诊。因此，现今，没有任何一个单维度分析的“金标准”可定义所有疾病，诊断时既要恰当选择相应检测方法，也要对包括临床信息在内的各项检测结果进行全面评估和信息整合，尤其在检测结果完全不一致或临床与病理不符时，才能最终进行合理解释和解读，进而给出最终综合诊断。因此，将现有诊断报告[5]进行多维度整合形成血液病理综合诊断报告就成为了一种必然。

迄今对血液病理整合报告格式尚未形成共识，但应至少包含以下要素：①基本信息：包括患者年龄、性别、取材部位、临床疑诊、送检科室、取材时间等；②标本大体情况：如大小、长短、切面质地等；③镜下形态学描述：应详尽，重点突出疾病特点；④其他实验室检查结果：包括特殊染色、免疫组化、流式免疫分型、遗传学及分子生物学等检查结果；⑤诊断结果：应将临床信息及病理相关检查结果进行整合，按照国际标准/规范出具最终综合诊断结果，可包括必要的讨论、参考文献及相关的建议；⑥医师签名及报告时间。最终呈现“一个患者、一个诊断、一份报告”。
